# Factors associated with self‐management among Vietnamese adults with type 2 diabetes

**DOI:** 10.1002/nop2.158

**Published:** 2018-05-14

**Authors:** Tiet‐Hanh Dao‐Tran, Debra Anderson, Anne Chang, Charrlotte Seib, Cameron Hurst

**Affiliations:** ^1^ Centre for Work, Organisation, and Well‐being Griffith University Brisbane Australia; ^2^ University of Medicine and Pharmacy at Ho Chi Minh City Vietnam; ^3^ School of Nursing and Midwifery Griffith University Gold Coast Australia; ^4^ School of Nursing Queensland University of Technology Brisbane Australia; ^5^ Clinical Epidemiology and Biostatistics Faculty of Medicine Chulalongkorn University Bangkok Thailand

**Keywords:** self‐efficacy, self‐management, type 2 diabetes

## Abstract

**Aim:**

The study described diabetes self‐management (DSM), diabetes knowledge, family and friends’ support, healthcare providers’ support, belief in treatment effectiveness and diabetes management self‐efficacy, and explored DSM's associations among Vietnamese adults with type 2 diabetes mellitus (T2DM).

**Design:**

A cross‐sectional design was applied.

**Methods:**

The study used self‐report questionnaires to collect data from 198 participants. Descriptive statistics and structural equation modelling (SEM) was used for data analysis.

**Results:**

Vietnamese adults with T2DM performed DSM limitedly in certain aspects. They had strong belief in treatment effectiveness, good family and friends support, limited diabetes knowledge, healthcare professional support and self‐efficacy. Their DSM was directly associated with diabetes knowledge, family and friends’ support, healthcare providers’ support, belief in treatment effectiveness and diabetes management self‐efficacy. Their DSM was indirectly associated with diabetes knowledge and family and friends’ support through their belief in treatment effectiveness and diabetes management self‐efficacy.

## 1 INTRODUCTION

Type 2 diabetes mellitus (T2DM), commonly known as adult onset diabetes, may lead to several complications (Huang et al., 2014). T2DM may also increase healthcare costs (Shah, Shamoon, Bikkina, & Kohl, 2017), reduce working years (von Bonsdorff et al., 2018) and lead to disability and death (Wu et al., 2016). The exact causes of T2DM are still unclear; however, this health condition can be managed with diabetes management activities such as healthy diet, regular physical activities, medication adherence, frequent blood glucose level self‐monitoring, weight control, tobacco avoidance and other preventive measures (American Diabetes Association, 2015).

Diabetes self‐management is an “active and flexible process where people with diabetes cooperate with healthcare professionals and other significant people to develop goals and strategies for their diabetes management and to perform the above diabetes management activities” (Yin, Savage, Toobert, Wei, & Whitmer, 2008). Because adults with T2DM usually manage their health outside formal healthcare settings, diabetes self‐management (DSM) has an important role in their health and well‐being, and so understanding DSM and its associations is important to promote health and well‐being for people with T2DM.

The prevalence of diabetes in Vietnam has increased five times over the last four decades (Pham & Eggleston, 2016), yet, knowledge about DSM and its association in this target population is still limited. The current study aimed to describe DSM, diabetes knowledge, family and friends’ support, healthcare providers’ support, belief in treatment effectiveness and diabetes management self‐efficacy and to explore DSM's associations among adults with T2DM in Vietnam.

## 2 BACKGROUND

Based on the triadic reciprocal interaction among personal factors, environmental factors and behaviour, Social Cognitive Theory (SCT) indicates that people will have little reason to convince themselves to change their current behaviours to healthy behaviours if they lack knowledge about the risks from the behaviours they enjoy and the benefits from a behaviour change (Bandura, 2004). In addition, people may not successfully change behaviours when they do not have the requisite knowledge and skills (Bandura, 2004). SCT also indicates that behavioural change will be easier if there are facilitators; however, if impediments exist, they may influence the process of behavioural change (Bandura, 2004). SCT further posits that personal and environmental factors influence behaviours through the psychological mechanisms of the self‐system, such as outcome expectation and self‐efficacy (Bandura, 2004). People are also more motivated to modify their behaviours when they believe these behaviours will leadto desirable health outcomes (Bandura, 2004). SCT indicates that when people have a high level of self‐efficacy to perform a specific task, they are more likely to initiate the task and put more effort into doing the task and are more likely to persist with the task in the face of difficulties (Bandura, 2004).

Diabetes knowledge is a general understanding about diabetes and diabetes management (Fitzgerald et al., 1998). Family and friends’ support is assistance that family and friends provide for patients’ diabetes management (Glasgow, Strycker, Toobert, & Eakin, 2000). Healthcare professionals’ support is the assistance that they provide for the patients’ diabetes management (Glasgow et al., 2005). Belief in treatment effectiveness is the belief that performing diabetes management activities are important for controlling blood glucose levels and for preventing complications from diabetes (Xu, Toobert, Savage, Pan, & Whitmer, 2008). Diabetes management self‐efficacy is patients’ confidence that they can undertake diabetes management activities (Sturt, Hearnshaw, & Wakelin, 2010).

For adults with type 2 diabetes, diabetes knowledge (Asmamaw, Asres, Negese, Fekadu, & Assefa, 2015; Islam et al., 2015; Kueh, Morris, Borkoles, & Shee, 2015; Yang et al., 2016), family and friends’ support, healthcare providers’ support (Hyman, Shakya, Jembere, Gucciardi, & Vissandjée, 2017; Mohn et al., 2015) and belief in treatment effectiveness (Xu et al., 2008) are associated with DSM. The content of diabetes knowledge is required to perform diabetes management activities (Yang et al., 2016). Specifically, adults with T2DM require knowledge about the importance of following DSM guidelines and appropriate ways of performing DSM activities (Kueh et al., 2015). Individualized knowledge improves diabetes management practices more than general knowledge does (van der Heide et al., 2014). Adults with T2DM are also less likely to engage in DSM when they lack social support (Koetsenruijter et al., 2016; Luo et al., 2015). Medication adherence is better among adults having partners (Lindsay, Natalie, Yael, Juan, & Jenny, 2018). Family and friends may provide information, hands‐on assistance and encouragement for adults with T2DM to perform DSM (Carolan, Holman, & Ferrari, 2015). Healthcare professionals can provide expert medical recommendations and collaborate with adults with T2DM to develop goals and strategies for diabetes management (Heisler, Cole, Weir, Kerr, & Hayward, 2007). With the support from healthcare providers, patients are more likely to engage in their self‐management (Alvarez, Greene, Hibbard, & Overton, 2016).

Diabetes management self‐efficacy is an important facilitator for performing DSM activities (Gunggu, Thon, & Whye Lian, 2016; Kueh et al., 2015; Tharek et al., 2018). Self‐efficacy had moderate association with self‐management (Tharek et al., 2018). The relationship between diabetes management self‐efficacy and DSM is significant, regardless of the patient's race or health literacy (Sarkar, Fisher, & Schillinger, 2006). Sarkar et al. (2006) found that increased in diabetes management self‐efficacy scores, people with T2DM increased appropriate diet, exercise, foot care and self‐monitoring blood glucose levels. Later, Xu et al. (2008) revealed that belief in treatment effectiveness and diabetes management self‐efficacy were mediators for the relationship between diabetes knowledge, healthcare providers’ communication, family support and DSM.

This study addressed these following research questions:

Research question 1: What are the levels of DSM, diabetes knowledge, family and friends support, healthcare providers’ support, belief in treatment effectiveness and diabetes management self‐efficacy of Vietnamese adults with T2DM?

Research question 2: What are the conceptual and mathematical associations among DSM and diabetes knowledge, family and friends support, healthcare providers’ support, belief in treatment effectiveness and diabetes management self‐efficacy among Vietnamese adults with T2DM?

Based on SCT and previous literature, this study hypothesized that among Vietnamese adults with T2DM:

Hypothesis 1: DSM is directly associated with diabetes knowledge, family and friends’ support, healthcare providers’ support, belief in treatment effectiveness and diabetes management self‐efficacy.

Hypothesis 2: The associations between DSM and diabetes knowledge, family and friends’ support and healthcare providers’ support would be mediated by belief in treatment effectiveness and diabetes management self‐efficacy.

## 3 METHOD

### 3.1 Design

A cross‐sectional design was applied to this study as the design allowed the study to describe variables and their associations at a concurrent time (Carlson & Morrison, 2009), which suited the study's aims and questions. In addition, this design only required a one‐off contribution (Carlson & Morrison, 2009), which was quite inexpensive and so increased the study's feasibility.

### 3.2 Setting, participants and procedure for data collection

This study reported data from 198 outpatients aged 18 years and older with T2DM for at least 6 months in a diabetes outpatient clinic of the tertiary practice and training hospital in the South of Vietnam. For powerful statistical analysis, the sample size was calculated based on the formula proposed by Kline (2015) for structural equation modelling (SEM), which states that 10 participants should be included for each free parameter. There are 18 parameters and thus the required sample size was 180. To account for 10% of potential missing data, 198 participants were chosen.

The clinic had approximately 200 outpatient visits a day. The clients were from different rural and urban areas in Vietnam. Random sampling was performed based on time. Doctors and nurses in the clinic selected potential participants who satisfied the inclusion criteria and introduced the research every hour. Each working day, the first round of data collection was at 10 a.m. and the last round was at 3:00 p.m. Research assistants supported the participants with an integration of the research participation process into their healthcare service. Nursing academics further explained the research if required and performed data collection. Data collection was conducted until the sample size was achieved. Details about the research setting, participants and procedure for data collection were also published elsewhere (Dao‐Tran, Anderson, Chang, Seib, & Hurst, 2017)**.**

### 3.3 Measurement instruments

This study used interview‐administered valid self‐report scales to measure the study variables. The instruments were selected if they had been validated in previous published studies and were able to comprehensively measure the scope of the study variables. Permissions to use the scales as applicable were gained prior to a use.

To measure DSM, the Diabetes Self‐Management Instrument was used (Lin, Anderson, Chang, Hagerty, & Loveland‐Cherry, 2008). This scale has 35 Likert‐scale questions. The scale asks participants to indicate the frequency with which they perform DSM activities. The answers are rated from 1 (*never*) to 4 (*always*) and the total possible scores ranged from 35‐140, with a higher score indicating a better DSM. The instrument has five subscales: self‐integration (e.g. “I can participate in social activities and still manage my diabetes”), self‐regulation (e.g. “I decide what action to take based on the results of my previous actions”), interaction with healthcare professionals and other significant people (e.g. “I am comfortable telling my health care providers how much flexibility I want in my treatment plan”), self‐monitor blood glucose levels (e.g. “When I feel unwell but I am not sure if the cause is either high or low blood glucose, I check my blood glucose levels as soon as possible”) and adherence to recommended regime (e.g. “I take my diabetes medication at the times prescribed”). This instrument was tested on 634 adults with T2DM in Taiwan and had a Cronbach's alpha coefficient of 0.94 and a test–retest correlation of 0.73 (Lin et al., 2008). The Vietnamese version of this scale had a Cronbach's alpha coefficient of 0.9 (Dao‐Tran et al., 2017).

To measure diabetes knowledge, the Diabetes Knowledge Test was used (Fitzgerald et al., 1998). This scale has 14 items, asking participants about their understanding of diabetes and diabetes management (e.g. “What effect will an infection most likely have on blood glucose? a. Lowers it, b. Raises it and c. Has no effect”). The scale asks participants to choose the best answer for each question. For each correct answer, they score 1 point and the possible scores range from 0‐14 with an average of 7; a higher score indicates a higher level of diabetes knowledge. If the participant has a score below 7, it suggests a low level of diabetes knowledge. The instrument was tested on 811 adults with diabetes in the USA and had a Cronbach's alpha coefficient of 0.71 (Fitzgerald et al., 1998). The Vietnamese version of this scale had a Cronbach's alpha coefficient of 0.7 (Dao‐Tran, 2013).

To measure family and friends’ support, The Family and Friends’ Support Scale was used (Glasgow et al., 2000). The scale has 8 Likert‐scale items. The scale asks participants to indicate the amount of support they have from their family and friends for their chronic disease management activities (e.g. “Have family and friends encouraged you to do the things you need to do for your illness?”). The answers are rated from 1 (not at all) ‐ 5 (a great amount) and the total possible scores range from 8‐40, with a higher score indicating more family and friends’ support received for chronic disease management activities. This instrument was tested on 123 patients with chronic disease in the USA and had a Cronbach's alpha coefficient of 0.75 and a test–retest correlation at 1 month of 0.72 (Glasgow et al., 2000). The Vietnamese version of this scale had a Cronbach's alpha coefficient of 0.9 (Dao‐Tran, 2013).

To measure healthcare providers’ support, the Patient Assessment of Chronic Illness Care was used. The scale has 20 Likert‐scale items. The scale asks participants to indicate the frequency with which they have experienced support from their healthcare providers for chronic disease management activities (e.g. “When I received care for my chronic illness over last 6 months, I was asked my idea when made a treatment plan”). The answers are rated from 1 (*almost never*) to 5 (*almost always*) and the total possible scores ranged from 20 to 100, with a higher score indicating a higher frequency of healthcare professionals’ support for participants for their chronic disease management activities. This instrument was tested on 336 patients with T2DM in the USA and had a Cronbach's alpha coefficient of 0.96 and a test–retest correlation over 3 months of 0.58 (Glasgow et al., 2005). The Vietnamese version of this scale had a Cronbach's alpha coefficients of 0.9 (Dao‐Tran, 2013).

To measure belief in treatment effectiveness, the Belief in Treatment Effectiveness Scale (Yin et al., 2008) was used. This scale has nine Likert‐scale items asking participants to indicate their level of beliefs about the importance of performing DSM behaviours for controlling blood glucose levels and for preventing complications (e.g. “How important do you feel that diabetic diet is for controlling blood glucose levels?”). The answers are rated from 1 (*not important at all*) to 5 (*extremely important*) and the total possible scores range from 9 to 45, with a higher score indicating a stronger belief in treatment effectiveness. The instrument was tested on 30 adults with T2DM in China and had a Cronbach's alpha coefficient of 0.81 (Yin et al., 2008). The Vietnamese version of this scale had Cronbach's alpha coefficients of 0.64–0.8 (Dao‐Tran, 2013).

To measure diabetes management self‐efficacy, the Diabetes Management Self‐efficacy Scale was used (Sturt et al., 2010). This scale has 15 Likert‐scale items. The scale asks participants to rate the level at which they feel confident to perform DSM activities (e.g. “I am confident that I am able to choose correct food”). The answers are rated from 1 (cannot do at all) ‐ 10 (certain can do) and the total possible scores range from 0 ‐ 150, a higher score indicating stronger confidence in performing diabetes management activities. A minimum score for a possible change in diabetes management behaviours was 106. The instrument was tested on 175 people with T2DM in the UK and had a Cronbach's alpha coefficient of 0.89 and a test–retest correlation at 1 month of 0.77 (Sturt et al., 2010). The Vietnamese version of this scale had a Cronbach's alpha coefficient of 0.7 (Dao‐Tran, 2013).

### 3.4 Data analysis

The SPSS (Statistical Package for the Social Sciences) version 23 and AMOS (Analysis of Moment Structures) version 23 were used for data entry and analysis (George & Mallery, 2016). As the continuous variables were not normally distributed, medians and ranges were used to describe the variables; the Spearman rho test was used to examine the associations between two continuous variables (George & Mallery, 2016).

The assumptions of approximately linear relationships, normal distribution and outliers were checked prior to performing SEM. As the study continuous variables were not normal distributed, the assumptions of parametric SEM were violated. Therefore, the asymptotically distribution‐free estimation and Bollen‐Stine bootstrap function were used rather than maximum likelihood was used in structural equation modelling statistical method to model the associations among the study variables (Kline, 2015). The model was fit if it satisfied the following criteria: (1) a non‐significant χ^2^ test; (2) 1 < χ^2^/*df* <2 and (3) root mean square error of approximation (RMSEA) <0.05 and *p* of close fit (PCLOSE) >.05; goodness‐of‐fit index (GFI) >0.9; (4) adjusted goodness‐of‐fit index (adjusted GFI) >0.9; (5) standardized root mean square residual (SRMR) <0.06 (Kline, 2015). The level of significance was 0.05.

### 3.5 Ethical considerations

This study received Research Ethics Committee approval from the researcher's University Research Ethics Committee (1100000448). Acceptance to collect data was received from the hospital. Besides sending the potential participants the study information and the consent forms, the researcher also verbally emphasized to potential participants that if they had any concern about participating, they had the right to refuse to participate or to stop participating at any time. Written consents were gained prior to data collection. No questionnaires contained any identification details but were allocated a code. As the study was conducted at a tertiary hospital, if the participants encountered any distress because of their participation, they could receive help from the hospital at the researcher's cost.

## 4 RESULTS

### 4.1 Description

In total, we invited 297 participants and 198 participants accepted to participate and completed the questionnaires (response rate = 66.7%). They reported frequent performance of DSM (median = 98, range = 49–140 out of a possible 140). In detail, they reported a good frequency of “adherence to recommended regimen” (median = 12, range = 3–12, out of a possible 12), of self‐integrating diabetes management activities into their regular lives (self‐integration median = 30, range = 13–40, out of a possible 40) and of “self‐regulation” (median = 27, range = 9–36, out of a possible 36). However, they reported limited “interaction with healthcare professionals and other significant people” (median = 18, range = 9–36, out of a possible 36) and low frequency of “self‐monitoring blood glucose levels” (median = 7, range = 4–16, out of a possible 16).

Of 198 participants, more than half (52.5%, *N *=* *104) could not answer 50% of the items about diabetes knowledge correctly. Adults with T2DM who participated in the study had good family and friends’ support (median = 31, range 20–96). Their healthcare providers’ support scores were generally low (median = 33.0, range 20–96). About three‐fifths of participants (59.6%, *N *=* *118) had scores for diabetes management self‐efficacy less than 106, which was the minimum score for a possible change in diabetes management behaviours. Overall, the participants had strong belief in treatment effectiveness (median = 41, range (19–45; Table 1).

**Table 1:** Diabetes knowledge, belief in treatment effectiveness, diabetes management self‐efficacy, family and friends ‘support, healthcare providers’ support among adults with type 2 diabetes in Vietnam (*N *=* *198)

	Possible scores	Median	Range
1. Diabetes knowledge	0–14	6	1–11
2. Belief in treatment effectiveness	19–45	41	19–45
3. Diabetes management self‐efficacy	15–150	99	25–150
4. Family and friends support	8–40	31	8–40
5. Healthcare providers’ support	20–100	33	22–96

### 4.2 Testing hypotheses

Findings from the literature review were used to develop a preliminary hypothesized model. To finalize the hypothesized model, the researcher examined the correlation among variables for adding the significant paths and removing the non‐significant paths between variables in the preliminary hypothesized model. The correlation matrix (Table 2) showed neither a significant relationship between diabetes knowledge and healthcare professionals’ support, nor a significant relationship between healthcare professionals’ support and diabetes management self‐efficacy. Therefore, these non‐significant paths were removed from the preliminary hypothesized model.

**Table 2:** Bivariate correlation matrix among study variables (*N *=* *198)

Study variables	1	2	3	4	5	6
1. Diabetes knowledge	1	0.33^b^	0.40^b^	0.27^b^	0.02	0.43^b^
2. Belief in treatment effectiveness		1	0.55^b^	0.40^b^	0.18^a^	0.52^b^
3. Diabetes management self‐efficacy			1	0.44^b^	0.13	0.66^b^
4. Family and friends’ support				1	0.23^b^	0.47^b^
5. Healthcare providers’ support					1	0.39^b^
6. Diabetes self‐management						1

a Correlation is significant at the 0.05 level (2‐tailed).

b Correlation is significant at the 0.01 level (2‐tailed).

**Figure 1:**
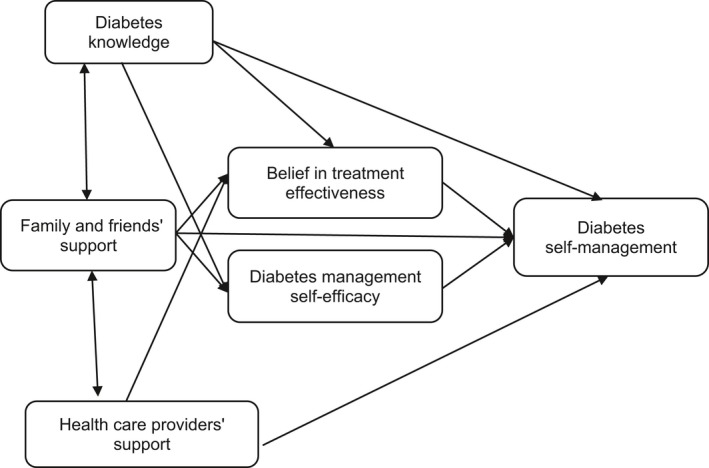
Hypothesized model of associations among the study variables

The final hypothesized model was then tested for a goodness of fit with the data. As the study variables were not normally distributed (*p* < .05), the assumptions of parametric SEM were violated. Therefore, asymptotically distribution‐free estimation was used in the data analysis. Figure 1 presents the results of the test of goodness of fit of the final hypothesized model against the data.

**Figure 2:**
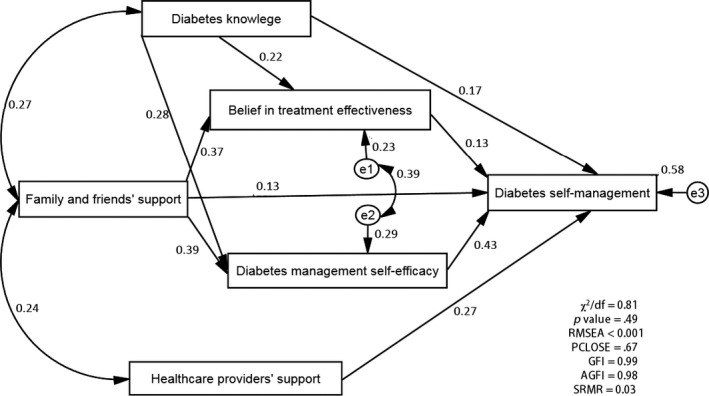
Final model of associations among the study variables

Based on the goodness‐of‐fit indices, the hypothesized model did not fit the data. The model was thus modified to achieve a sufficient fit. The final model given in Figure 2 achieved the desired goodness‐of‐fit value for the data (χ^2^/*df* = 0.81; *p *=* *.49; RMSEA <.001; PCLOSE = 0.67; GFI = 0.99; adjusted GFI = 0.98; SRMR = 0.03). Figure 2 demonstrates that diabetes knowledge (standardized β = 0.17, *p *<* *.001), family and friends’ support (standardized β = 0.13, *p *<* *.001), healthcare providers’ support (standardized β = 0.27, *p *<* *.001), diabetes management self‐efficacy (standardized β = 0.43, *p *<* *.001) and belief in treatment effectiveness (standardized β = 0.13, *p *<* *.01) directly influenced DSM. The numbers near the single‐headed arrows are standardized betas, whereas those proximal to the double‐headed curved arrows represent correlation coefficients. The results also indicated that belief in treatment effectiveness and diabetes management self‐efficacy only mediated the influences of diabetes knowledge and family and friends’ support on DSM among adults with T2DM in Vietnam (*p *<* *.05). Among the factors investigated, diabetes management self‐efficacy appeared to have the strongest direct (largest standardized β) influence on DSM (standardized β = 0.43, *p *<* *.001).

## 5 DISCUSSION

This study explored DSM and its associations among adults with T2DM in Vietnam. The study found that generally the participants reported quite high involvement in the integration of diabetes management in their daily life, regulation of daily life with diabetes management and adherence to the recommended regimen. By self‐monitoring blood glucose levels, people with diabetes can make better decisions for their diabetes management interventions; however, this practice was rarely found among the study participants. A possible explanation is that participants do not recognize the important of frequently self‐monitoring of their blood glucose levels. In addition, the cost of a self‐monitoring machine and glucose test sticks are high for the average income of this population and therefore were not available for many of the participants. The study participants also reported low frequency of interacting with healthcare professionals and other significant people to manage their health condition. This study was conducted at a tertiary hospital where healthcare professionals see approximately 200 patients in the clinic every day. This does not allow sufficient time for effective communication between healthcare professionals and clients. On the other hand, participants may have believed that diabetes is their own health condition and that they should control it by themselves. They may also have believed that other people do not understand the condition and may provide inappropriate interventions or diabetes management pressures. These are possible explanations for why the study participants do not ask for support from other people. However, these are speculations and further research is warranted.

Regarding DSM's associations, this study found that adults with T2DM had limited diabetes knowledge, good family and friends’ support, very limited healthcare professionals’ support, inadequate diabetes management self‐efficacy and strong belief in treatment effectiveness. In addition, the study found that there was a positive direct impact of diabetes knowledge on DSM. This means that when adults with T2DM have more diabetes knowledge, they are more likely to perform DSM in better ways for their health. This findings is consistent with findings from previous studies (Asmamaw et al., 2015; Kueh et al., 2015; van der Heide et al., 2014; Yang et al., 2016) and SCT (Bandura, 2004). To enhance the likelihood of DSM being practised among adults with T2DM in Vietnam, healthcare professionals need to advise their clients of the importance of monitoring their blood glucose levels and the meaning of A1C (Yang et al., 2016). Healthcare professionals in Vietnam also need to provide advice on appropriate DSM activities (Kueh et al., 2015) and individualize their advice for diabetes management (van der Heide et al., 2014). Research has shown that for each rise in score in the diabetes knowledge scale, there are increases in adherence to the diabetes diet (*OR* 1.23, 95% CI [1.10, 1.38]), blood glucose self‐measurement (*OR* 1.29, 95% CI [1.13, 1.48]) and regular exercise (*OR* 1.15, 95% CI [1.03, 1.28]) (Persell et al., 2004). As presented, SCT indicates that people have little reason to convince themselves to change behaviours they enjoy if they lack knowledge about the health risks and benefits from a certain health behaviour (Bandura, 2004). They are also unlikely to follow desirable behaviours if they do not have the requisite knowledge or means to use them (Bandura, 2004).

Also in line with findings from previous research, this study found support from healthcare providers and family and friends directly influenced the DSM of adults with T2DM (Koetsenruijter et al., 2016; Luo et al., 2015). This finding is also consistent with SCT, which proposes that facilitators may influence the process of behaviour change and people will be more likely to change behaviours when they have a supportive environment (Bandura, 2004). Applying this principle, family, friends and healthcare professionals may influence DSM behaviours by providing those with T2DM with appropriate instruments, information and emotional support (Carolan et al., 2015).

Moreover, the study found a positive direct impact of diabetes management self‐efficacy and belief in treatment effectiveness on DSM. This means that when adults with T2DM are more confident in performing DSM activities and believe more strongly in the effectiveness of diabetes management, they are more likely to modify their DSM behaviours to achieve a better health outcome. This finding is consistent with previous studies (van der Heide et al., 2014; Xu et al., 2008) and SCT (Bandura, 2004). As presented, SCT indicates that people will be more motivated to modify their behaviours when they believe these behaviours will lead to desirable health outcomes and when people possess a high level of self‐efficacy to perform a task, they will be more likely to perform the task (Bandura, 2004).

With SEM, this study also found an indirect impact on diabetes knowledge and family and friends’ support on DSM via belief in treatment effectiveness and diabetes management self‐efficacy (Xu et al., 2008). These findings are also consistent with those of a previous study (Xu et al., 2008) and SCT (Bandura, 2004). Using SEM statistics, Xu et al. (Xu et al., 2008) revealed that diabetes management self‐efficacy was a mediator for the relationship between diabetes knowledge, family support and DSM. Bandura (2004) also indicated that the psychological mechanisms of the self‐system, such as belief in treatment effectiveness and self‐efficacy, can mediate the impact of personal and environmental factors on behaviour.

Summing up the direct and indirect impact, self‐efficacy appeared to be the factor most influencing DSM. This finding is consistent with a previous study's findings and SCT (Bandura, 2004). Xu et al. (2008) found that self‐efficacy not only had a direct impact on DSM but also mediated the impact of diabetes knowledge and family and friends’ support on DSM and thus it became the factor that had the strongest influence on DSM among adults with T2DM in China. In SCT, self‐efficacy is a core factor in providing the foundation for behavioural achievement and people with a high level of self‐efficacy will be more likely to initiate a task and complete it even in the face of difficulties (Bandura, 2004).

Interestingly, one finding that was inconsistent with the study by Xu et al. (Xu et al., 2008) and SCT (Bandura, 2004) was that SCT postulates that healthcare professionals’ support influences DSM through self‐efficacy and belief in treatment effectiveness (Bandura, 2004), yet, the findings did not show an association between healthcare professionals’ support and diabetes self‐efficacy, or a belief in treatment effectiveness. One possible explanation is that the target group received limited support from their healthcare providers and therefore a significant relationship between healthcare providers’ support and diabetes management self‐efficacy and belief in treatment effectiveness could not be detected because of our modest sample size, or because of the limited spectrum of healthcare professional support captured in our sample.

### 5.1 Strengths and limitations

Regarding the limitations, first, the use of face‐to‐face interviews to collect data may have introduced some reporting bias. However, the researcher and her data collection assistants were not in hospital uniform during the data collection phase and data collection was conducted in a private area in an effort to limit the patients from over‐ or under‐reporting. Second, since a cross‐sectional design was applied, findings about relationships among variables could not be concluded as causal. Third, even though the participants were chosen randomly from the hospital, they were not chosen randomly from the Vietnamese population and so these findings cannot be generalized to the broader Vietnamese population. Finally, only some of the factors potentially influencing DSM among adults with T2DM in Vietnam, which may themselves be endogenous, were investigated. Other factors—for example, education level (Xu et al., 2008), work characteristics (Xu et al., 2008) and health status (Chlebowy, Hood, & LaJoie, 2010)—may also influence DSM among adults with T2DM in Vietnam, but were not included in the model in this study.

However, this study had several strengths. First, it contributed to a comprehensive description about factors associated with DSM for the first time in a Vietnamese T2DM population. This exploration therefore allowed healthcare professionals in Vietnam to gain a better understanding of their population's DSM and thus they may be able to provide better support for people with T2DM in the future. Second, most previous studies in T2DM populations have focused on patients’ adherence to recommendations of DSM behaviours, but this study acknowledges and accounts for the patients’ collaboration with healthcare professionals and other significant people (Lin et al., 2008). Thus, the researcher emphasized the importance of the patients’ roles in partnerships for managing their health conditions, rather than solely by their adherence to medical recommendations (Lin et al., 2008). Third, the data were collected through Vietnamese versions of valid instruments, which were tested for internal consistency reliabilities (Dao‐Tran, 2013). This improved the reliability of the findings. Fourth, as the data were collected through interview‐administered self‐report questionnaires, missing data were minimized. Finally, as SEM was used for data analysis, both direct and indirect influences among variables were fully explored (Kline, 2015).

### 5.2 Recommendations for practice and future research

Supported self‐management is required for adults with type 2 diabetes (Captieux et al., 2017). At present, interventions for diabetes management in Vietnam focus solely on diabetes knowledge and have a limited emphasis on other factors such as diabetes management self‐efficacy and collaboration between patients with healthcare professionals and other significant people. The findings suggest that to improve DSM among adults with T2DM in Vietnam, a stronger focus on diabetes management self‐efficacy and healthcare professionals’ support, as well as diabetes knowledge, is important. Interactive‐ and theoretical‐based intervention is recommended for the effectiveness (Cheng et al., 2017; Zhao, Suhonen, Koskinen, & Leino‐Kilpi, 2017). Healthcare providers should not only provide health education to improve diabetes knowledge for their patients (Kueh et al., 2015; van der Heide et al., 2014), they should also need to discuss diabetes management plans with their patients and allow their patients to take part in making decisions about their diabetes management plans (Heisler et al., 2007). Family and friends should also be encouraged to participate in the health education activities to gain more diabetes knowledge so they are able to provide appropriate support for their family member with T2DM. In addition, peer support activities should be applied, in consultation with experts (Baumann, Frederick, Betty, Jospehine, & Agatha, 2015). This would allow the patients to receive encouragement from others in their ability to perform DSM and to see others like themselves able to perform DSM and experience success in performing their own DSM activities. As a result, they will be more confident in their ability to perform DSM.

Use of technology is another recommendation for supporting self‐management among adults with T2DM (Petersen & Hempler, 2017). The app may provide information about diabetes knowledge and how to self‐manage via game activities (Petersen & Hempler, 2017). The app may also save the medical record for monitoring and making decision about their diabetes management (Petersen & Hempler, 2017). In addition, as monitoring blood glucose is important for diabetes management (Claude Mbanya, Aschner, Chan, Jose Gagliardino, & Saji, 2017), using colour indicating self‐monitor blood glucose handset may also be useful as it provides a quick interpretation of the blood test results and may indirectly improve their blood glucose control (Oliver, Gerd, Bettina, Rosa Maria, & Michael, 2017).

For future studies, to determine whether the relationships revealed are causal, a cohort design is recommended. In addition, a more comprehensive model of factors with variables such as education level, health status and work‐related factors should be examined in larger samples across the Vietnamese population.

## 6 CONCLUSION

Vietnamese adults with T2DM performed DSM inactively. They have limited diabetes knowledge, good family and friends’ support, very limited healthcare professionals’ support, a strong belief in treatment effectiveness and inadequate diabetes management self‐efficacy. Self‐efficacy has the strongest influence on DSM. Diabetes knowledge and family and friends’ support directly and indirectly influences DSM through belief in treatment effectiveness and diabetes management self‐efficacy. Findings from this study suggest that interventions to promote diabetes knowledge, healthcare providers’ support and diabetes management self‐efficacy are required to improve DSM among Vietnamese adults with T2DM in the future. While currently healthcare providers are providing traditional health education to enhance patients’ diabetes knowledge, future interventions might also include interventions to enhance healthcare providers’ support and patients’ self‐efficacy. SCT has been shown here to be likely to provide benefits in guiding these future interventions. Findings from this study may be applied to similar populations as this study's participants, including adults with type 2 diabetes in Asian nations and Vietnamese adults with T2DM internationally.

## CONFLICT OF INTERESTS

No conflict of interest exists.

## AUTHOR CONTRIBUTIONS

THDT, DA, AC: Study design; THDT: Data collection; THDT, CH: Data analysis; THDT, CH, DA, AC, CS: Manuscript writing. All authors read and approved the manuscript.
